# Supplementary material for: Denosumab and Zoledronic Acid Differently Affect Circulating Immune Subsets: A Possible Role in the Onset of MRONJ
Source: Cells. 2023 Oct 11;12(20):2430. doi: 10.3390/cells12202430 (PMC10605172; doi:10.3390/cells12202430)
Supplement: Supplementary file 1 [file cells-12-02430-s001.zip › cells-2628604-supplementary.pdf]

Figure S1

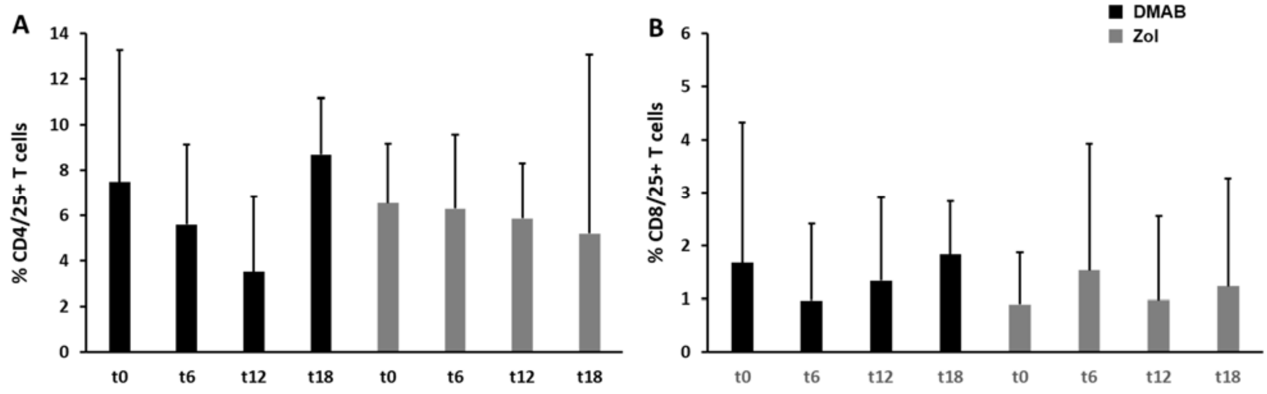

Figure S1. CD25 expression on CD4+ Th and CD8+ Tc cells.

No significant variations were observed in T cells for CD25 expression at any time nor with Dmab and Zol.

Figure S2

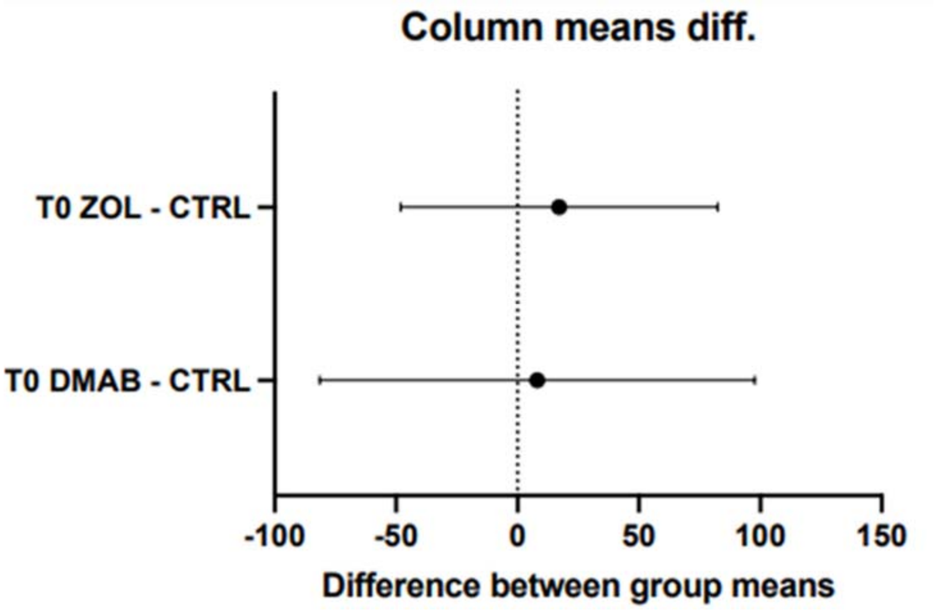

Figure S2. Cytokines levels in control group and Dmab or Zol patients are comparable.

At T0, the level of cytokines in sera of patients with neo-diagnosis of breast cancer (CTRL) did not show significant differences compared with yet treated breast cancer patients, before starting Dmab or Zol.
